# Band Anti-Crossing Model in Dilute-As GaNAs Alloys

**DOI:** 10.1038/s41598-019-41286-y

**Published:** 2019-03-26

**Authors:** Justin C. Goodrich, Damir Borovac, Chee-Keong Tan, Nelson Tansu

**Affiliations:** 1 Center for Photonics and Nanoelectronics, Department of Electrical and Computer Engineering, Lehigh University, Bethlehem, PA 18015 USA; 2 Department of Electrical and Computer Engineering, Clarkson University, Potsdam, NY 13699 USA

## Abstract

The band structure of the dilute-As GaNAs material is explained by the hybridization of localized As-impurity states with the valance band structure of GaN. Our approach employs the use of Density Functional Theory (DFT) calculated band structures, along with experimental results, to determine the localized As-impurity energy level and coupling parameters in the band anti-crossing (BAC) *k ∙ p* model for N-rich alloys. This model captures the reduction of bandgap with increasing arsenic incorporation and provides a tool for device-level design with the material within the context of the *k ∙ p* formalism. The analysis extends to calculating the effect of the arsenic impurities on hole (heavy, light and split-off) effective masses and predicting the trend of the bandgap across the entire composition range.

## Introduction

Major breakthroughs in solid-state lighting and energy efficiency applications have been enabled in recent decades through thorough investigation of the class of III-nitride semiconductors, including GaN, InN, AlN, and their alloys with one another^1,2^. Advancements in both material epitaxy methods, along with new, innovative approaches for device level design, have enabled the production of highly efficient light emitting diodes (LED) devices^3,4^. Refined understanding of intrinsic material properties of this semiconductor family has enabled their use in various applications, such as high-speed electronics, lasers, and solar energy applications^5–7^.

A key feature of the III-nitride semiconductors is their direct bandgap property, which is required for use in energy-efficient light-emitting applications. The materials also possess other useful optoelectronic, chemical, and tribological properties, which allow them to be applied in a wide variety of devices and applications^8,9^. In contrast to the substantial work towards investigating the family of III-nitride semiconductors, with prime examples being the InGaN and AlGaN alloys^10^, much less literature has been published on the dilute-anion III-nitride semiconductor alloys. Recent work has illustrated the potential of using the dilute-arsenic GaNAs alloy for achieving a broad range of bandgaps across the entire visible spectrum^11–13^. Analysis also indicates that the material is promising due to its reduced Auger recombination rate in comparison to GaN^14^, making it a strong candidate for use in future optoelectronic applications.

The first reported incorporation of arsenic into a GaN crystal was reported by Li and co-workers via the metalorganic chemical vapor deposition (MOCVD) growth technique^12^. Incorporation of ~6.7% As-content via MOCVD was later reported by Kimura and co-workers^13^. Additionally, GaNAs alloys across the entire As-composition have been grown using the molecular beam epitaxy (MBE) technique by Yu and co-workers^15–17^. These experiments indicate that the GaNAs material exhibits an increasing reduction of the bandgap from that of GaN with higher arsenic incorporation, indicating an experimental pathway towards tunability of the bandgap and other electronic properties by controlling the impurity incorporation. Thus, as implementing the GaNAs material into future device design may prove important, an accurate description of the band structure in the context of the ***k ∙ p*** perturbation theory for device-modeling purposes is instrumental.

Previous studies have shown that the band anti-crossing (BAC) model can be applied as a technique to specifically describe the band structures of highly-mismatched semiconductor alloys^18^. Specifically, the band anticrossing model has been widely used in the ***k · p*** method to investigate the electronic properties of highly-mismatched alloyed semiconductors, including III-V alloys such as dilute-N GaAsN and InGaAsN^19,20^, dilute-N GaPN^20^, dilute-N AlGaNAs^21^, and others^22–24^, along with various IV-class semiconductors^25^, and II-VI semiconductors^26–28^. The various models used for different material systems involve modifying the Hamiltonian to introduce localized energy levels associated with the dilute-ion incorporation of a new element into the host semiconductor material. As part of the highly-mismatched semiconductor alloy class, the BAC model can be potentially useful for analyzing the band structure of the dilute-As GaNAs alloys. Developing the band anticrossing model for dilute-As GaNAs alloys would provide a key step towards understanding the band structure evolutions of the alloy and providing a more accurate band structure analysis for device design purpose in the future. Using DFT results to fit our model allows for the atomistic description of the electronic states in the material to be transformed into useful parameters for nanoscale device level design.

In this work, we develop a valence-band hybridization BAC model to determine the characteristics of the electronic structure and properties for dilute-As GaNAs alloys. The BAC parameters (*C*_*GaNAs*_ and *E*_*As*_) of the electronic structure of the GaN_1−x_As_x_ material system with As-content ranging from 0% up to 12.5% are evaluated and presented. The effect of the BAC interaction on the band dispersions of the GaN_1−x_As_x_ alloys are analyzed. Our findings indicate the validity of using valence-band hybridization BAC model to explain the significant electronic band structure modifications in dilute-As GaNAs alloys when compared to the GaN binary alloy.

## Computational Method

A 6-band Hamiltonian from ***k ∙ p*** perturbation theory is commonly used to model the valence band electronic structure of binary wurtzite semiconductors^29^. The eigensolutions of this Hamiltonian are doubly degenerate and split into 3 distinct energy bands, known as the heavy hole (HH), light hole (LH), and split-off (SO) bands. The energy dispersions of these bands are calculated as a function of wave vector near the gamma point (***k*** = 0). Use of the correct material parameters provides an accurate description of the band structure at this band edge (near Γ-point), which can then be used in self-consistent analysis to calculate the spontaneous emission rate, gain properties, and other important characteristics of the resultant quantum well structures^30^.

**Figure 1:**
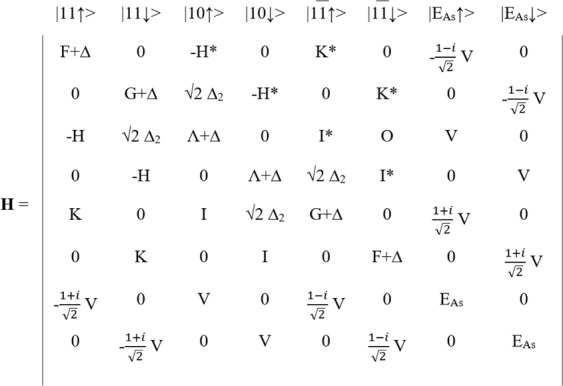
Valence band hybridization BAC Hamiltonian for N-rich GaNAs semiconductors.

For the ternary dilute-anion GaNAs material system, the traditional 6-band ***k ∙ p*** Hamiltonian used to model the valence band of GaN is extended by including two additional states that represent the localized impurity level energy *E*_*As*_, as shown in Fig. 1. This is known as the valence band anticrossing model^31^. The localized energy state is used to modify the original GaN host energy bands with band edges set using the virtual crystal approximation (VCA) between GaN and GaAs. The hybridization energy *V*, which describes how strongly the localized state perturbs the host states, is given by the hybridization energy parameter *C*_*GaNAs*_ and assumes a square-root dependence on the impurity concentration. More remote states are neglected due to their large distances from the valence band maximum of GaN.

In the Hamiltonian from Fig. 1, the parameters can be defined using the following equations:

1 $$\begin{array}{c}{\rm{\Delta }}={E}_{v}(x)-[{{\rm{\Delta }}}_{1}(x)+{{\rm{\Delta }}}_{2}(x)]\,\end{array}$$

2 $$\begin{array}{c}F={{\rm{\Delta }}}_{1}(x)+{{\rm{\Delta }}}_{x}(x)+\,\lambda +\theta \,\end{array}$$

3 $$\begin{array}{c}G={{\rm{\Delta }}}_{1}(x)-{{\rm{\Delta }}}_{x}(x)+\,\lambda +\theta \end{array}$$

4 $$\begin{array}{c}\,\lambda ={A}_{1}{k}_{z}^{2}+{A}_{2}{k}_{t}^{2}+{D}_{1}{\varepsilon }_{zz}+{D}_{2}({\varepsilon }_{xx}+{\varepsilon }_{yy})\end{array}$$

5 $$\begin{array}{c}\theta ={A}_{3}{k}_{z}^{2}+{A}_{4}{k}_{t}^{2}+{D}_{3}{\varepsilon }_{zz}+{D}_{4}({\varepsilon }_{xx}+{\varepsilon }_{yy})\end{array}$$

6 $$\begin{array}{c}H=i{A}_{6}{k}_{z}{k}_{t}-{A}_{7}{k}_{t}\end{array}$$

7 $$\begin{array}{c}I=i{A}_{6}{k}_{z}{k}_{+}+{A}_{7}{k}_{+}\end{array}$$

8 $$\begin{array}{c}K={A}_{5}{k}_{+}^{2}\end{array}$$

9 $$\begin{array}{c}V={C}_{GaNAs}\sqrt{x}\end{array}$$

where the wave vector terms are represented as:

10 $$\begin{array}{c}{k}_{t}=\sqrt{{k}_{x}^{2}+{k}_{y}^{2}}\end{array}$$

11 $$\begin{array}{c}{k}_{+}={k}_{x}+i{k}_{y}\end{array}$$

and the virtual crystal approximation terms from eqs (1–3) are:

12 $$\begin{array}{c}{E}_{v}(x)=({E}_{g,GaN}-{E}_{g,GaAs})\cdot x\end{array}$$

13 $$\begin{array}{c}{{\rm{\Delta }}}_{1}(x)={{\rm{\Delta }}}_{1,GaN}\,\ast \,(1-x)+\,{{\rm{\Delta }}}_{1,GaAs}\cdot x\end{array}$$

14 $$\begin{array}{c}{{\rm{\Delta }}}_{2}(x)={{\rm{\Delta }}}_{2,GaN}\,\ast \,(1-x)+{{\rm{\Delta }}}_{2,GaAs}\cdot x\end{array}$$

The material parameters used in the relations above are presented in Table 1. The bandgaps and band edge (crystal field and split-off) energies of the binary endpoint materials, GaN and GaAs, are used in the virtual crystal approximation terms. The virtual crystal approximation assumes a linear interpolation of the band edges between the binary materials. The effective mass parameters are not interpolated; the parameters for GaN are used for analyzing the perturbative effect arsenic concentration has on band dispersions and effective mass.

**Table 1:** Material parameters used for GaN and GaAs. Parameters taken from refs^11,30,35,37^.

Parameters	GaN	GaAs
**Energy parameters**		
E_g_ (eV)	3.645	1.424
Δ_cr_ = Δ_1_ (eV)	0.010	0
Δ_so_/3 = Δ_2_ = Δ_3_ (eV)	0.00567	0.133
**Effective mass parameters**		
A_1_	−7.21	
A_2_	−0.44	
A_3_	6.68	
A_4_	−3.46	
A_5_	−3.40	
A_6_	−4.90	
A_7_	0	

The eigenenergies of the Hamiltonian in Fig. 1 are evaluated at the gamma (Γ) point as a function of *x* (As-content) and the BAC parameters (*E*_*As*_ and *C*_*GaNAs*_). Diagonalizing the Hamiltonian reveals a restructuring of the valance band into four doubly-degenerate bands, denoted as *E*_*As−like*_, *E*_*A−like*_, *E*_*B−like*_, and *E*_*C−like*_, respectively. The first band, *E*_*As−like*_, is a newly formed energy band by the interaction of the arsenic states with the host GaN states, whereas the other bands correspond to the GaN-perturbed HH, LH, and SO bands, respectively. For *x* > 0, *E*_*As−like*_ emerges as a new valence band maximum (VBM), and the fundamental bandgap of the material is denoted as the difference in energy between the conduction band minimum energy of GaN and this new VBM. One can then find the optimal set of BAC parameters that reach a minimum of the error between the BAC bandgap as a function of *x* versus a separate theoretical calculation or experimental measurement of the bandgap.

Suitable BAC parameters for the dilute-As GaNAs ternary alloy system can be found by comparing the BAC band gap to that of First-Principle Density Functional Theory (DFT) calculations. First-Principles DFT calculations were performed by using the Projector Augmented Wave (PAW) method using the Local Density Approximation (LDA) exchange-correlation potential in the MedeA VASP software. The scissor operator was used to correct for errors in the bandgap calculation^32^. Although the use of the LDA is known to introduce some errors into the band structure calculations^33,34^, particularly in the effective masses, only the bandgap as a function of arsenic content is used on our calculation. Supercells of gallium and nitrogen atoms were developed with a substitution of a single As on an N site, thereby changing the GaN supercell into a GaNAs supercell. Details of the computational method and results can be found in previous publications^11^.

## Results and Discussion

**Figure 2:**
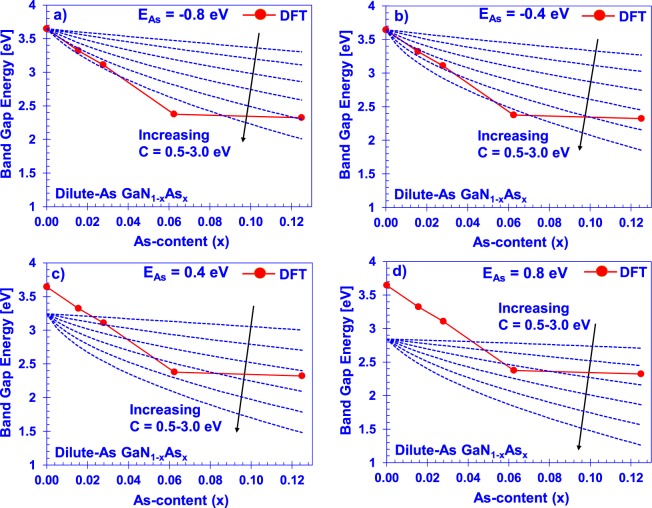
Arsenic impurity parameter variation of the dilute-As GaNAs material with *E*_*As*_ set to (**a**) −0.8 eV, (**b**) −0.4 eV, (**c**) 0.4 eV, and (**d**) 0.8 eV with respect to the GaN VBM, presented alongside the DFT-reduction of bandgap.

The localized energy *E*_*As*_ was varied from −0.8 eV to 0.8 eV in 0.01 eV increments while the hybridization energy *C*_*GaNAs*_ was varied from 0 eV to 3 eV in the same increments. These were used as fitting parameters to minimize the error between the DFT-calculated band gaps and the resultant bandgaps from the diagonalized Hamiltonian. Figure 2(a–d) show selected BAC parameter sweeps of the ***k ∙ p****-*modelled reduction of energy bandgap against the DFT calculations. The effect on the bandgap reduction of the four *E*_*As*_ values are shown. This comparison shows that the *E*_*As*_ value for the dilute-As GaNAs is lying slightly below the valence band maximum of the GaN.

**Figure 3:**
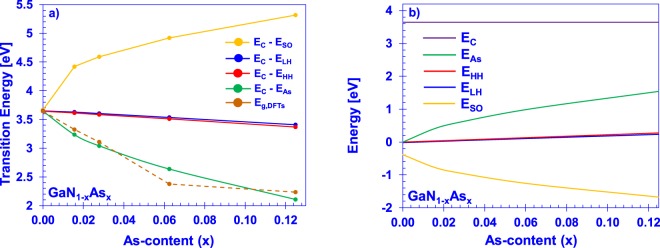
Best-fit valence (**a**) subband transition energies and DFT-calculated reduction of band gap and, (**b**) subband positions, at the gamma point via the BAC model using parameters *E*_*As*_ = −0.39 eV and *C*_*GaNAs*_ = 2.57 eV.

The dilute-As GaNAs material system is best-modelled with the parameters of localized impurity energy at *E*_*As*_ = −0.39 eV (below the VBM of GaN) and a coupling constant *C*_*GaNAs*_ = 2.57 eV. The BAC-calculated subband transition energies at the gamma point with these energy parameters are presented in Fig. 3. An interesting feature revealed in the transition energies in Fig. 3 is the drastic increase of the split-off energy as a function of *x*. The model predicts large split-off energies (>1 eV), compared to both that of GaN^30^ and GaAs^35^, at even low levels of arsenic incorporation (~1.56%). This prediction is supported by the DFT-calculations^11^, which also indicate a large splitting of the split-off band from the heavy hole and light hole bands in the dilute-As regime.

**Figure 4:**
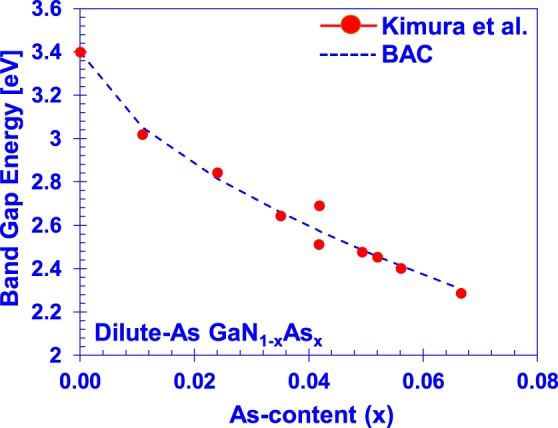
Best-fit reduction of bandgap energy to MBE-grown samples by Kimura *et al. E*_*As*_ = −0.32 eV, *C*_*GaNAs*_ = 2.64 eV.

The previously described method of determining the BAC parameters was also performed on experimental measurements of the bandgap of epitaxial GaNAs films reported by Kimura *et al*.^13^ This dataset is very-well fit by the BAC model, using the same GaN and GaAs parameters as those in Table 1, except the different value of GaN band gap of *E*_*g*,*GaN*_ = 3.39 eV, as measured in the experiment. Figure 4 presents the best fit found using the BAC parameters *E*_*As*_ = −0.32 eV and *C*_*GaNAs*_ = 2.64 eV. Both coupling constants (*E*_*As*_ and *C*_GaNAs_) are in close agreement with those obtained via the DFT calculations, within a value of 0.1 eV between the DFT and experimental results. This provides assurance that these values of the parameters reasonably model the reduction bandgap in the N-rich valence band hybridization BAC model.

Previous work on the N-rich GaN_1−x_As_x_ valence band hybridization BAC model done by Wu *et al*.^31^ suggested BAC parameters of *E*_*As*_ = 0.62 eV and *C*_*GaNAs*_ = 0.75 eV. These values differ drastically from our results and predict a positive *E*_*As*,_ placing the newly-formed valence subband to be above the VBM of GaN. Moreover, an important aspect of our parameters is that they accurately predict the bandgap in the dilute limit (as *x* → 0) to approach that of GaN (~3.4 eV). As indicated in Fig. 2, impurity energy levels above the VBM will cause the bandgap calculated from the Hamiltonian to approach the value of *E*_*g*,*GaN*_ − *E*_*As*_ in the dilute limit. Use of localized energies below the VBM resolves this issue while providing a good fit to the reduction of the bandgap in the target composition range.

The discrepancy between the values reported in this report and the previous work can be potentially explained by the nature of the growth conditions and how the arsenic atoms are incorporated into the alloy. It is known that the fabrication of GaNAs alloys by MBE is highly dependent upon the growth conditions. Arsenic-doped gallium nitride (*x* ~ 0.002) samples grown under gallium-rich conditions exhibit a broad PL at around 2.6 eV (~0.8 eV below that of GaN), whereas films grown under nitrogen-rich conditions (with the same arsenic flux) exhibit a much smaller shift from the gallium nitride peak to a value around 3.34 eV (less than 0.1 eV from GaN)^36^. This fact, in conjunction with the fact that the DFT-calculated bandgaps of the minimally clustered, single crystal material approaches GaN in the dilute limit, suggests that it is necessary to achieve high nitrogen to gallium ratios to promote crystalline GaNAs alloy growth. The bandgap of alloys grown under gallium-rich conditions may be attributed to transitions involving vacancy defects rather than being a transition associated with a true alloy state. Further experimental and theoretical analysis into the nature of arsenic-doped GaN and phase-separation in dilute-As GaNAs films is necessary to understand the nature of the bandgap in this very dilute limit.

A prediction of the bandgap across a greater composition range of the ternary alloy GaN_1-x_As_x_ from GaN (*x* = 0) to GaAs (*x* = 1) can be achieved by using a quasi-linear interpolation between the valence BAC model on the dilute-anion side and the conduction BAC model on the dilute-cation side^31^. This interpolation is achieved using the equation:

15 $$\begin{array}{c}{E}_{g}(x)=(1-x)\cdot {E}_{g,N-rich}(x)+x\cdot {E}_{g,As-rich}(x)\end{array}$$

**Figure 5:**
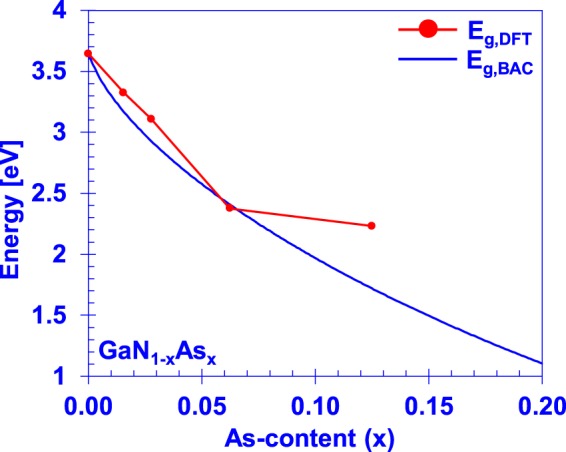
Crystalline GaN_1−x_As_x_ bandgap extended over the wurtzite-crystal composition range using a quasilinear weighting between the GaN and GaAs BAC models.

Such an interpolation, through the regime in which the alloy is expected to be in wurtzite configuration with minimal-clustering, is presented in Fig. 5 using our DFT-calculated valence BAC parameters of *E*_*As*_ = −0.39 eV, *C*_*GaNAs*_ = 2.57 eV, and known conduction BAC parameters of *E*_*N*_ = 1.63, *E*_*GaAsN*_ = 2.7 eV^15–17^.

**Figure 6:**
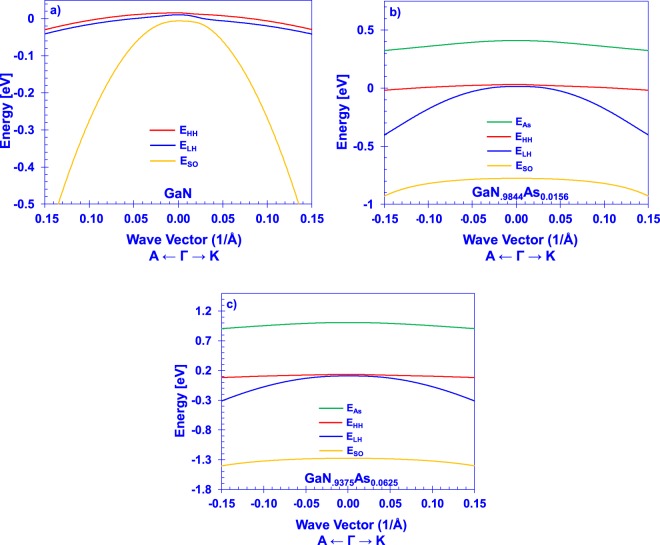
***k ∙ p*** band dispersions of (**a**) GaN, (**b**) GaN_0.9844_As_0.0156_, and (**c**) GaN_0.9375_As_0.0625_.

By inserting wave vector dependence into the Hamiltonian, the effect of the arsenic impurity on the band dispersions can be determined. The valence subband dispersions of unstrained GaN were calculated by the 6-band ***k ∙ p*** model, without the valence BAC effect, using the parameters for the material listed in Table 1. Furthermore, the valence subband dispersions of GaN_0.9844_As_0.0156_ and GaN_0.9375_As_0.0625_ were determined from the 8-band valence BAC ***k ∙ p*** model using the DFT-fit BAC parameters and the GaN effective mass parameters from Table 1. These dispersions are presented in Fig. 6.

It is evident that the valence band structure of the GaNAs alloy is heavily perturbed by the presence of the localized impurity. Most notable is the existence of a new, relatively heavy valence band maximum that originates from the *E*_*As−like*_ eigenstate, despite the use of the *E*_*As*_ localized energy below the valence band maximum of GaN. The transition between this state and the conduction band of GaN is responsible for the fundamental bandgap of the alloy. A strong interaction occurs between this state and the GaN split-off band, resulting in a large deepening of the split-off band energy at even low arsenic concentrations, along with an increase of the effective mass of this subband. The heavy hole and light hole bands remains close in energy at the gamma point, increasing in energy slowly according to the virtual crystal approximation. Nevertheless, the character of these dispersions demonstrates a strong interaction with the arsenic-state, rapidly deviating from the form predicted by the standard 6-band model for wurtzite semiconductors. This indicates that the use of the band anti-crossing model in device-model applications can have profound deviations from using a more simplistic 6-band valence band model of GaNAs which assumes no deviation in the wave vector dependence.

**Figure 7:**
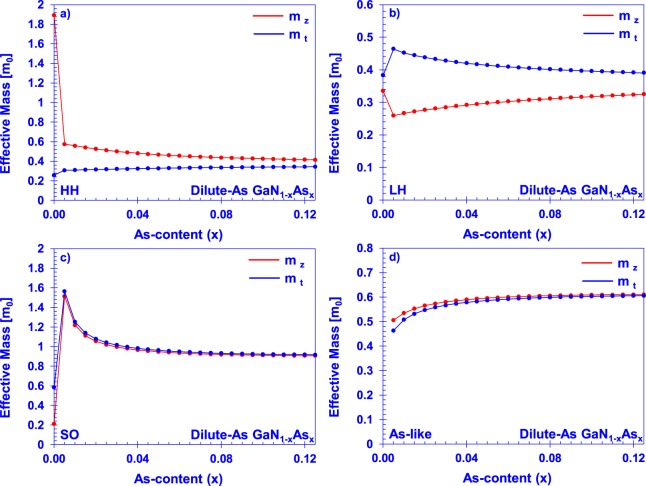
GaN_1−x_As_x_ valence subband effective masses in A and K directions as a function of arsenic content from ***k ∙ p*** for the (**a**) heavy hole, (**b**) light hole, (**c**) split-off, and (**d**) arsenic-like energy bands.

Near the gamma point, energy bands can be modeled with an effective mass, which describes the approximate parabolic behavior of the energy well when wave vector is near a minimum. Calculations of the effective mass for each of the valence subbands were performed by determining the difference in energy between the subband at the gamma point and at very small ***k***-space increments in the **A** (*k*_*z*_) and **K** (*k*_*x*_) directions. This was performed using the 6-band model for GaN and the 8-band valence BAC model for GaN_1−x_As_x_ in increments of *x* = 0.005 to *x* = 0.125, corresponding to the maximum arsenic incorporation of 12.5% investigated in the DFT calculation. The effective masses for each of the subbands in both directions are plotted in Fig. 7.

There is a discontinuity between the nature of the GaNAs subband effective masses from the BAC model in the very dilute limit to those of GaN from the standard 6-band model, even when the same effective mass parameters are used in both Hamiltonians. This is due to the very strong interaction of the arsenic impurity at even very low incorporation levels, along with the formulation of the Hamiltonian itself. Further investigation, either theoretical or experimental, into the nature of the band dispersions in this dilute limit will be useful for refining the material parameters.

## Conclusions

In conclusion, a band anti-crossing model has been developed for dilute-As GaNAs material system with As-content up to 12.5%. BAC parameters required for the band anticrossing model are determined by comparing the results from the model with the DFT-calculation findings and experimental findings. The BAC parameters are used in the valence band hybridization ***k ∙ p*** perturbation theory to model the effect of an arsenic impurity on the GaN host crystal. Use of a localized impurity energy (*E*_*As*_) below the valence band minimum of GaN is found to well-model the reduction of bandgap for the material, with the important feature of converging to the bandgap of GaN in the dilute limit. These parameters are essential for device-level modelling by providing a method to capture the key electronic properties of the alloy, allowing for the detailed design and analysis of green-emitting III-nitride LEDs and other optoelectronic structures based upon this alloy.
